# NKL-Code in Normal and Aberrant Hematopoiesis

**DOI:** 10.3390/cancers13081961

**Published:** 2021-04-19

**Authors:** Stefan Nagel

**Affiliations:** Department of Human and Animal Cell Lines, Leibniz-Institute DSMZ, German Collection of Microorganisms and Cell Cultures, Inhoffenstr. 7B, 38124 Braunschweig, Germany; sna@dsmz.de; Tel.: +49-531-2616-167

**Keywords:** NKL, homeobox, oncogene, T-ALL

## Abstract

**Simple Summary:**

Gene codes represent expression patterns of closely related genes in particular tissues, organs or body parts. The NKL-code describes the activity of NKL homeobox genes in the hematopoietic system. NKL homeobox genes encode transcription factors controlling basic developmental processes. Therefore, aberrations of this code may contribute to deregulated hematopoiesis including leukemia and lymphoma. Normal and abnormal activities of NKL homeobox genes are described and mechanisms of (de)regulation, function, and diseases exemplified.

**Abstract:**

We have recently described physiological expression patterns of NKL homeobox genes in early hematopoiesis and in subsequent lymphopoiesis and myelopoiesis, including terminally differentiated blood cells. We thereby systematized differential expression patterns of eleven such genes which form the so-called NKL-code. Due to the developmental impact of NKL homeobox genes, these data suggest a key role for their activity in normal hematopoietic differentiation processes. On the other hand, the aberrant overexpression of NKL-code-members or the ectopical activation of non-code members have been frequently reported in lymphoid and myeloid leukemia/lymphoma, revealing the oncogenic potential of these genes in the hematopoietic compartment. Here, I provide an overview of the NKL-code in normal hematopoiesis and instance mechanisms of deregulation and oncogenic functions of selected NKL genes in hematologic cancers. As well as published clinical studies, our conclusions are based on experimental work using hematopoietic cell lines which represent useful models to characterize the role of NKL homeobox genes in specific tumor types.

## 1. Hematopoiesis

**Figure 1:**
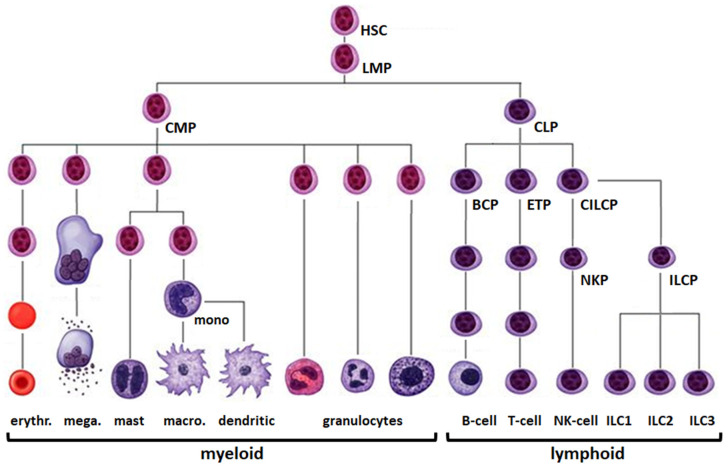
Schematic presentation of hematopoiesis. This diagram depicts the progenitors and terminally differentiated cells of hematopoiesis, comprising the myeloid (**left**) and lymphoid (**right**) lineage. The following abbreviations are used: BCP: B-cell progenitor, CILCP: common ILC progenitor, CLP: common lymphoid progenitor, CMP: common myeloid progenitor, ETP: early T-cell progenitor, HSC: hematopoietic stem cell, ILC(P): innate lymphoid cell (progenitor), LMP: lymphoid and myeloid primed progenitor, NKP: NK-cell progenitor. Figure modified from www.pinterest.de.

The hematopoietic system comprises all cells derived from hematopoietic stem cells (HSCs) (Figure 1). Novel comprehensive sequencing approaches have extended the characterization and derivation of progenitors and mature blood cells [1,2,3]. Hematopoietic stem and progenitor cells generate common myeloid and lymphoid progenitors (CMPs and CLPs), founders of the myeloid and lymphoid lineages, respectively. CLPs produce all types of lymphocytes comprising B-cells, T-cells, natural killer (NK)-cells and innate lymphoid cells (ILCs). Early B-cell development takes place in the bone marrow and begins with the CLP-derived B-cell progenitor (BCP). BCPs differentiate into naïve B-cells via the pro-B- and pre-B-cell stages. Naïve B-cells migrate from the bone marrow into lymph nodes, spleen and other lymphoid tissues to undergo final differentiation into memory B-cells and plasma cells via the stage of germinal center (GC) B-cells. In contrast, CLP-derived early T-cell progenitors (ETPs) migrate into the thymus to complete their differentiation via the double negative (DN) and double positive (DP) stages into CD4 and CD8 single-positive T-cells. CMPs produce all types of myeloid cells including erythrocytes and granulocytes which comprise eosinophils, neutrophils and basophils. Intermediate differentiation steps towards mature granulocytes include granulocyte macrophage progenitors (GMPs), early and late pro-myelocytes, and meta-myelocytes. Further myeloid cells are megakaryocytes and mast cells in addition to monocytes, which are able to differentiate into macrophages or dendritic cells. Unlike lymphocytes, all types of myeloid cells develop within the bone marrow.

The main regulatory steps of hematopoiesis operate at the transcriptional level by several transcription factors (TFs) identified as master genes for the development of early hematopoiesis, lymphopoiesis or myelopoiesis [3,4]. TAL1 and LYL1 are basic helix-turn-helix TF proteins and regulate vital steps in early and late hematopoiesis [5]. GATA factors are zinc-finger proteins that control hematopoietic stem cells (GATA2), erythropoiesis (GATA1), and NK- and T-cell development (GATA3) [6,7]. Additional TFs controlling specific processes in hematopoietic differentiation are EBF1 and PAX5 (development of B-cells), BCL11B (T-cells), NFIL3 (NK-cells), ID2 (ILCs), and SPI1/PU.1 (myeloid cells) [8,9,10,11,12]. These developmental regulators belong to different protein families, demonstrating that various types of TFs mediate the transcriptional control of hematopoiesis.

## 2. Classification of Homeobox Genes

**Figure 2:**
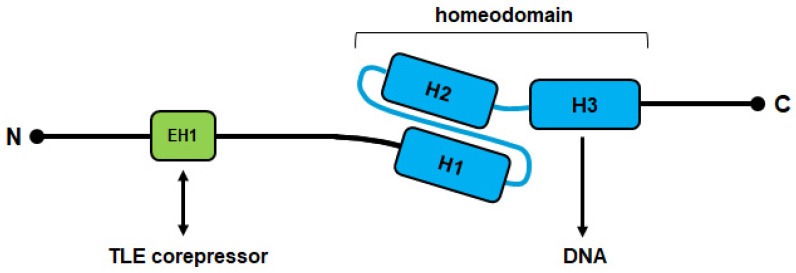
Schematic structure of NKL homeodomain proteins. N: N-terminal end; C: C-terminal end; EH1: conserved engrailed homology domain consisting of about 8 amino acid residues (shown in green); the conserved homeodomain consists of 60 amino acid residues which generate three helices (H1-3) and is shown in blue; the N- and C-terminal parts show no sequence conservation. The EH1 domain and the homeodomain interact with particular components of the gene regulatory machinery including corepressors of the TLE family and specific DNA sequences, respectively.

Homeobox genes encode one of the largest groups of TFs in the human genome [13]. Generally, they regulate basic development and differentiation in both embryogenesis and the adult. These genes share the conserved homeobox which is 180 bp long and encodes the homeodomain at the protein level. This domain consists of 60 amino acid residues which form three helices, generating a specific 3D structure. The homeodomain performs specific interactions with DNA, chromatin, non-coding RNAs, and cooperating TFs, thus representing the core of their gene regulatory activities [14]. Specific DNA contacts are realized by helix 3 which fits into the major groove [15]. The remaining helices stabilize the domain structure and, together with flanking amino acid residues, allow additional DNA interactions (Figure 2).

From the systematic classification of all 235 human homeobox genes emerged a panel of eleven classes and several subclasses. The main classes are named antennapedia (ANTP) and paired box (PRD). Other classes identified are CERS, CUT, HNF, LIM, POU, PROS, SINE, TALE, and ZF. According to this system, NKL homeobox genes represent a subclass of the ANTP class and number 48 members in humans [16].

NK-like homeobox genes were first reported by Nirenberg and Kim (abbreviated as NK) in the fruit fly Drosophila, later summarized as NKL. In this developmental model organism, the NKL subclass members are arranged in a cluster consisting of six genes [17]. Additional orthologous genes were later identified, extending this group of genes. Comparative genome analyses revealed this clustering to be the ancient gene order which remains barely discernible in vertebrates [18]. Thus, human NKL homeobox genes show only relicts of a clustered arrangement while the HOX genes are still present in a clustered order in both fruit fly and humans.

NKL proteins share a conserved homeodomain which display the amino acid tyrosine at position 54 [19]. In addition, NKL homeodomain proteins contain a short and conserved sequence in their N-terminal region which has been termed engrailed-homology motif (EH1) [20]. This EH1 sequence mediates physical interactions with corepressors of the Groucho-family (transducin-like enhancer of split, TLE), thus transforming NKL homeodomain factors into transcriptional repressors [21] (Figure 2). Most NKL homeobox genes are involved in mesodermal development, possibly recapitulating their ancient functions, including the repression of alternative differentiation lineages [22,23]. Many NKL homeobox genes operate as master factors, further demonstrating the regulatory potential of these genes. For example, NKX2-5 controls the development of the spleen and heart, and NKX2-1 that of the lung and thyroid gland [24,25].

## 3. NKL-Code in Hematopoiesis

**Figure 3:**
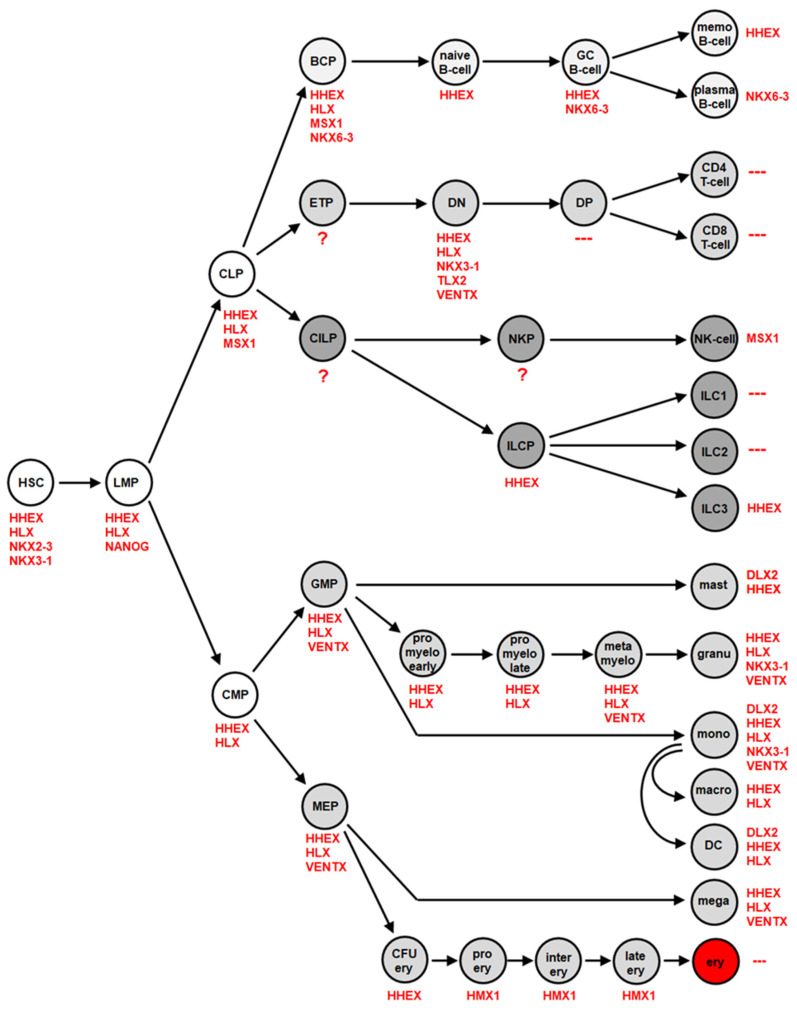
The NKL-code in hematopoiesis This diagram depicts activities of NKL homeobox genes during early hematopoiesis, in lymphopoiesis including the development of T-cells, B-cells, NK-cells and ILCs, and in myelopiesis. Each cell/stage is labelled with the accordingly expressed NKL homeobox genes. BCP: B-cell progenitor, CILP: common innate lymphoid progenitor, CLP: common lymphoid progenitor, CMP: common myeloid progenitor, DC: dendritic cell, DN: double negative, DP: double positive, ETP: early T-cell progenitor, GC B-cell: germinal center B-cell, GMP: granulocytic-monocytic progenitor, HSC: hematopoietic stem cell, ILC(P): innate lymphoid cell (progenitor), LMP: lymphoid and myeloid primed progenitor, memo B-cell: memory B-cell, MEP: megakaryocyte-erythroid-progenitor. Of note, this code was generated analyzing expression profiling and RNA-seq data.

In several studies, we examined the physiological expression pattern of NKL homeobox genes in stem cells, progenitors and terminally differentiated blood cells across the whole hematopoietic spectrum. The lymphoid lineage was analyzed in four studies using public datasets of developing and mature T-cells, B-cells, NK-cells and ILCs [26,27,28,29]. Datasets covering the myeloid lineage were analyzed in a single study [30]. The accumulated results are summarized in Figure 3, showing NKL homeobox gene activities in distinct hematopoietic stages and cell types. Altogether, eleven NKL homeobox genes were identified, comprising DLX2, HHEX, HLX, HMX1, MSX1, NANOG, NKX2-3, NKX3-1, NKX6-3, TLX2 and VENTX. Their unique expression pattern has been termed NKL-code [26,27,28,29,30].

The most prominent among these genes are HHEX and HLX, active in HSCs, multiple progenitors, and various mature blood cells. The expression of NKX2-3 and NANOG is restricted to the earliest stages of hematopoiesis, including HSCs and lymphoid and myeloid primed progenitors (LMPs), respectively. NKX6-3 is just expressed in the B-cell lineage, TLX2 in the DN-stage of T-cell development, DLX2 in mature mast cells and monocytes, and HMX1 in the course of erythropoiesis. MSX1 is expressed in CLPs and in NK-cells. NKX3-1 shows a diverse expression pattern comprising undifferentiated HSCs and DN T-cells in addition to differentiated granulocytes and monocytes. The absence of NKL homeobox gene activity was reported for DP, CD4 and CD8 single-positive T-cells and for ILC1 and ILC2. Thus, in contrast to these lymphoid cell types, the remaining hematopoietic cells specifically express one or several members of the NKL homeobox gene subclass. Of note, some screenings for gene activities in these various hematopoietic cell types were conducted by expression profiling. The used gene chips do not contain the complete panel of known human genes and lacked eleven NKL homeobox genes (Table 1). However, analysis of RNA-seq data generated from various hematopoietic stem and progenitor cells confirmed the inactivity of these eleven genes in immature stages [31], supporting the integrity of the described NKL-code.

These findings were promoted by data reported for particular NKL homeobox genes and hematopoietic cell types. HLX and HHEX represent the first described non-HOX homeobox genes expressed in hematopoietic cells [32,33]. Expression analyses of both genes revealed activity in B- and myeloid cells while T-cells tested negative [32,34,35,36]. Moreover, the downregulation of HHEX was shown to be crucial for normal T-cell differentiation and its activity was absent in plasma cells [36]. Analysis of HHEX-knockout mice showed the disturbed development of all types of lymphocytes, demonstrating the importance of HHEX for lymphopoiesis [37]. Recently, a role of HHEX was shown in the development of memory B-cells, its expression in accordance with the NKL-code [27,38]. The forced expression of HLX in hematopoietic progenitors enhanced myeloid differentiation but arrested the development of B-cells at the pro-B-cell stage and of T-cells at the DP stage, highlighting the shutdown of its activity for lymphocyte maturation [34,39]. According to the NKL-code, VENTX is active in erythroid progenitors and is silenced at early stages of erythropoiesis [30]. Consistently, VENTX drives the expansion of erythroid cells and suppresses genes, promoting the terminal differentiation of erythrocytes [40].

Homeobox genes regulate basic processes in tissue and organ development. This potential in addition to specific activities of closely related homeobox genes has been referred by the annotation of codes. The HOX-code describes the ordered expression of the clustered HOX genes along the anterio–posterio axis of the developing hindbrain and of the embryonal pharynx [41,42]. The DLX-code addressed the expression pattern of DLX genes along the dorsal–ventral axis in the pharyngeal region of the embryo [43]. The identity of the developing placodes which generate different sensory organs is manifested by the expression of particular PAX genes and, accordingly, is termed the PAX-code [44]. Finally, the TALE-code defines a signature of TALE class homeobox genes in lymphopoiesis [45]. Thus, the NKL-code along with other homeobox gene codes serves to outline and understand developmental gene activities and their subordinate differentiation processes.

## 4. NKL Homeobox Genes in Non-Hematopoietic Tissues

In addition to hematopoiesis, NKL-code genes are also expressed in the development of other tissues or organs. HHEX (hematopoietically expressed homeobox gene) was one of the first homeobox genes identified in blood cells [33]. However, the same study reported that the activity of this gene is not restricted to the blood cell compartment—it was also detected in the lung and liver. Accordingly, HHEX is involved in differentiation processes of the lung and liver in addition to the thyroid gland and pancreas development [46]. Interestingly, the other basic hematopoietic NKL homeobox master gene, namely HLX, is also involved in the development of the lung and liver, in addition to the gut [47,48].

NANOG was first identified as a prominent stem cell factor in embryonic stem cells and plays an important role in the generation of induced pluripotent stem cells (iPSC) [49,50,51]. Together with other stem cell factors, such as OCT4, SOX2, FOXD3 and KLF4, NANOG establishes a gene network to control stemness [52,53]. Neural crest cells are generated during vertebrate development at the neural plate border and differentiate into a plethora of cells and tissues. Therefore, these cells possess conspicuous stem cell potential [54]. NANOG regulates together with VENTX the development of migrating neural crest cells. Both genes represent an evolutionary innovation of vertebrates, establishing a multipotent progenitor state [55]. Recently, VENTX was identified as an essential gene in pluripotent stem cells [56], further supporting the stemness-mediating activity for these NKL homeobox genes.

MSX1 is also associated with neural crest development and expressed at the neural plate border, controlling the generation of neural crest cells [54]. In successive steps, MSX1 regulates the development of the craniofacial region and the central nervous system [57]. Additionally, MSX1 is a central player in odontogenesis. Accordingly, mutations in the MSX1 coding region are associated with craniofacial malformations and teeth abnormalities [58,59]. At all these sites, BMP-signaling mutually regulates MSX1 activity [60,61]. Interestingly, the ectopic expression of MSX1 in myotubes promotes the dedifferentiation of these immature muscle cells [62], demonstrating the developmental power of this NKL homeobox gene.

NKX3-1 plays a central role in prostate development. Accordingly, the downregulation or mutation of this gene is seen in prostate cancer, indicating tumor suppressor activity [63]. HMX1 regulates ear development. Patients with certain regulatory HMX1 mutations display malformations of the outer ear while DLX2 is expressed in, and plays a developmental role for, the inner ear [64,65]. Moreover, together with other DLX genes, DLX2 organizes the development of the pharyngeal region in the embryo [43].

Thus, although NKL homeobox genes control specific processes in differentiation, they are not restricted to one particular tissue or organ. Their activities are mostly connected with early developmental stages or the progenitor state. In mature cells, NKL homeobox genes may support the manifestation of a particular cell type. These observations may indicate that these differentiation factors perform their function by controlling basic lineage decisions and the context-dependent regulation of cell-type specific genes. Hence, the mutation or deregulation of these genes is connected with malformations, developmental disorders or hereditary diseases.

## 5. Deregulated NKL Homeobox Genes in Hematopoietic Malignancies

In cell and tissue differentiation, specific intermediate stages are distinguishable. In cancer, normal progression is disturbed and developmental arrest at particular immature stages is a dominant and widespread feature of malignant cells [66,67,68]. Thus, cancer fairly represents a developmental disease. Accordingly, the forced expression of NKL homeobox gene HLX in hematopoietic progenitors resulted in the developmental arrest of pro-B-cells [39]. This experiment was the first hint for the oncogenic potential of NKL homeobox genes in hematopoietic cells. Later, Ferrando and coworkers described the aberrant expression of the NKL homeobox gene TLX1 at the DP stage in T-cell acute lymphoid leukemia (T-ALL), indicating that this gene specifically promotes the developmental arrest of malignant thymocytes [69]. The strong correlation of aberrantly expressed NKL homeobox genes with particular stages of lymphoid differentiation highlights the developmental potency of these genes in hematopoietic tumors.

Table 1 summarizes deregulated NKL homeobox genes identified in hematopoietic malignancies. Accordingly, 35 aberrantly expressed genes of the 48 gene strong NKL homeobox gene subclass have been described in these cancers, demonstrating their ubiquity and oncogenic impact in the hematopoietic compartment. Indeed, despite their relatively recent discovery, NKL homeobox genes form the most substantial group of oncogenes in leukemia and lymphoma. With few exceptions, most deregulated NKL homeobox genes are expressed in both lymphoid and myeloid malignancies. The following account summarizes deregulating mechanisms and oncogenic functions of selected NKL homeobox genes hitherto described in hematopoietic tumors.

TLX1: In 1991, the investigation of chromosomal rearrangement t(10;14)(q24;q11) in T-ALL patients by the group of Stanley Korsmeyer demonstrated the juxtaposition of T-cell receptor gene TRD and T-cell leukemia homeobox 1 (TLX1, formerly HOX11) [70]. Since then, aberrantly activated TLX1 emerged as a hallmark oncogene for this malignancy, although TLX1 is expressed in merely 10% of pediatric and 30% of adult T-ALL patients [69]. Retrospectively, TLX1 was the first NKL homeobox gene reportedly deregulated in hematopoietic malignancies. Normally, TLX1 plays a fundamental role in early spleen development, though it is not expressed in hematopoietic cells [71]. It is believed that TLX1 may perform its oncogenic function by the reactivation of embryonal splenic activities.

TLX3: In 2001, the group of Roger Berger identified a novel cryptic translocation, t(5;14)(q35;q32), in T-ALL subsets. This aberration activates T-cell leukemia homeobox 3 (TLX3, formerly HOX11L2) by juxtaposition to the BCL11B locus and was detected in about 25% of pediatric and 5% of adult T-ALL patients [72]. Despite their high similarity, TLX1- and TLX3-rearranged T-ALL patients show different prognoses [73]. Functionally, T-cell differentiation factor BCL11B is downregulated by chromosomal juxtaposition with TLX3 or directly by TF TLX1 [74]. Furthermore, TLX1 and TLX3 interact with TF ETS1 in T-ALL cells, thereby blocking TCR-rearrangement and T-cell differentiation [75]. Another gene family member, TLX2 (HOX11L1), though physiologically expressed in T-cell progenitors, is only rarely deregulated in T-ALL [26].

NKX2-5: In 2003, we reported an alternative, cytogenetically identical t(5;14)(q35;q32) in two T-ALL cell lines which juxtaposes BCL11B with NK2 homeobox 5 (NKX2-5, formerly CSX1) [76]. This indicative finding helped us to uncover the wider oncogenic role of NKL homeobox genes in T-ALL, then comprising TLX1, TLX3 and NKX2-5. Today, 24 deregulated NKL homeobox genes are described in T-ALL [26,77]. However, NKX2-5 is rarely expressed in this malignancy [26,78]. Normally, NKX2-5 plays a key physiological role in the development of the spleen and heart [24,71]. Uniting its physiological and leukemic roles, myocyte-specific enhancer factor 2C (MEF2C) has been shown to serve as a target gene of NKX2-5 in both heart and leukemic T-cells [79,80]. MEF2C itself has, meanwhile, emerged as a major oncogene in T-ALL, alternatively activated by chromosomal deletion and translocation, or by other deregulated transcription factors [80,81,82].

NKX2-1: In T-ALL, chromosomal translocation t(7;14)(q34;q13) causes the aberrant activation of NK2 homeobox 1 (NKX2-1) [81]. NKX2-1, like NKX2-2 and NKX2-5, activates MEF2C in T-ALL [81]. In addition, NKX2-1 is aberrantly activated in diffuse large B-cell lymphoma (DLBCL) where it is deregulated by an altered chromatin configuration instead of a chromosomal rearrangement [83]. Thus, the same NKL homeobox gene is aberrantly activated by diverse mechanisms in different lymphoid malignancies. Normally, NKX2-1 plays a role in the development of the lung and thyroid gland but is absent from hematopoietic cells and tissues [25].

NKX3-1: NK3 homeobox 1 (NKX3-1, formerly BAPX2) is hematopoietically expressed in stem cells and T-cell progenitors [26]. In T-ALL, NKX3-1 is aberrantly activated by the TFs TAL1 and GATA3 [84]. In leukemic T-cells, NKX3-1 expression correlates with oncogenic TAL1 activity and additionally with the expression of homeobox gene SIX6 [85]. SIX6 is directly activated by NKX3-1 or alternatively by the closely related factor NKX3-2 (BAPX1) as described in both T-ALL patients and cell lines [86,87]. However, the leukemic role of SIX6 in T-ALL remains unclear.

MSX1: Muscle segment homeobox gene 1 (MSX1) is aberrantly expressed in several types of leukemia and lymphoma, including T-ALL, Hodgkin lymphoma (HL) and mantle cell lymphoma [26,88]. In the hematopoietic system, MSX1 is normally expressed in lymphoid progenitors and mature NK-cells, reflecting its contrasting roles as oncogene in T-ALL and tumor suppressor in NK-cell leukemia [28]. In T-ALL, the downregulated BMP-signaling pathway and upregulated chromatin-mediator AUTS2 have been reported as activating mechanisms for MSX1 [89,90]. MSX1 plays a major role in the generation and development of neural crest cells [54], supporting its role as lineage regulator in stem/progenitor cells.

HLX: H2.0-like homeobox (HLX) is overexpressed in several hematopoietic malignancies including HL, DLBCL and anaplastic large cell lymphoma (ALCL) [27,29]. Aberrant activation by STAT3 plays a dominant role in HLX expression in these tumor types. The requirement of the nuclear localization of STAT3 for HLX activation has been shown in HL cell line L-540 [91]. The role of the Epstein–Barr virus-mediated activation of STAT3 in HLX expression was analyzed in the DLBCL cell line DOHH-2 [92]. Finally, STAT3 represents a hallmark factor in ALCL [93]. Analyses of several ALCL cell lines revealed genomic gains at STAT3 and HLX loci and demonstrated a direct activating impact of STAT3 in HLX expression, highlighting HLX as important oncogene and STAT3-target in this malignancy [29].

NKX2-3: NK2 homeobox 3 (NKX2-3) is a member of the NKL-code and expressed in HSCs [26]. Its focused activity suggests a regulatory role in maintaining stemness and self-renewal. In splenic marginal zone lymphoma (SMZL) aberrant NKX2-3 expression is driven by chromosomal translocation t(10;14)(q24;q32) via juxtaposition to the IGH-locus [94].

NANOG: Like NKX2-3, NANOG belongs to the NKL-code and shows restricted activity in early stem and progenitor cells [26]. The aberrant expression of NANOG has been described for NKT-cell lymphoma, AML and MDS [30,95]. Using AML cell line NOMO-1 as a model, we revealed the NOTCH signaling pathway and TET2-mediated aberrant DNA-methylation in NANOG activation [30]. Micro-RNA host gene MIR17HG is a target of NANOG in both AML and neural stem cells, contributing to the oncogenic function of this homeobox gene [30,96].

DLX2: Distal-less homeobox 2 (DLX2) is physiologically expressed in mature myeloid cells including mast cells, monocytes and monocyte-derived dendritic cells [30]. DLX2 is located at chromosomal position 2q31 next to its paralogue gene DLX1, which is normally silent in hematopoietic cells. Aberrant expression has been reported for both DLX1 and DLX2 in T-ALL, AML and MDS patients, indicating divergent regulation in the malignant context [26,30]. In AML, oncogenic ERK-signaling drives the expression of both DLX1 and DLX2 [97].

HMX: The human genome contains three related HMX genes. HMX1 is located at chromosomal position 4p16 and expressed in early erythropoiesis while HMX2 and HMX3 are located side by side at 10q26, being silent in hematopoiesis [30]. However, HMX2 and HMX3 are aberrantly expressed in AML and the activity of HMX1 was detected in subsets of myelodysplastic syndrome (MDS) patients [30]. HMX2 and HMX3 show an absence of chromosomal rearrangements and are activated by IL7-signaling and via the mutation of two transcription factor-binding sites in an AML cell line (EOL-1). One mutation transforms an inhibitory NFkB-site into an activatory SP1-site while the other mutation generates an activating ETS-site [98]. Functional analyses revealed the inhibition of differentiation gene EPX and activation of the receptor HTR7 that drives oncogenic ERK-signaling [98]. Furthermore, HMX2 and HMX3 inhibit eosinophilic cell differentiation as shown by morphological changes in the AML cell line model [98].

Together, these examples show that deregulated NKL homeobox genes perform basic oncogenic functions in hematopoietic malignancies. NKL homeobox genes act as oncogenes and tumor suppressor genes in other tissues as well, such as NKX2-1 and NKX3-1 in lung and prostate carcinoma, respectively [63,99]. In leukemia and lymphoma, deregulating mechanisms include chromosomal translocation, chromatin modifications, mutations in TF binding sites, altered DNA-methylation, and aberrant activities of particular TFs and signaling pathways such as BMP, ERK, or NOTCH. Downstream effects of these genes are even more diverse, including the activation of specific TFs and micro RNAs, inhibitory interaction with cell type specific TFs, the deregulation of differentiation markers and signaling pathways, in addition to disturbed proliferation, apoptosis and differentiation. Notably, ectopically expressed NKL homeobox genes highlight the role of activated alternative developmental processes for this group of oncogenes. The NKL-code and its oncogenic violation discloses an underlying pathological principle and the list of oncogenic activities performed by deregulated NKL homeobox genes comprises all characteristics described for tumorigenesis.

## 6. Tumor Cell Lines as Models for Deregulated NKL Homeobox Genes

Cell lines are clonal cells which grow unlimited in culture. They are genomically stable, survive freezing and thawing, and are available from cell line banks. Tumor cell lines represent experimental models for that tumor type from which they were derived. Therefore, it is of fundamental importance to use authenticated and well characterized cell lines to be able to transfer cell line data to particular cancers. In the literature evaluated and systematically listed, hematopoietic cell lines have been described which meet these criteria and include the tumor types ALCL, AML, chronic myeloid leukemia, B-cell precursor ALL, double-hit B-cell lymphomas, HL, MDS, multiple myeloma, NK-cell leukemia, primary effusion lymphoma and primary mediastinal B-cell lymphoma [100,101,102,103,104,105,106,107,108,109,110,111,112]. Recently, we have generated exomes and transcriptomes for 100 leukemia/lymphoma cell lines, providing comparative sequence data for a large panel of hematopoietic tumor entities [113]. These data may help to identify novel genes involved in the pathogenesis of particular tumor types.

Deregulated NKL homeobox genes were usually first ascertained in patients and investigated experimentally in cell lines to reveal mechanisms of deregulation and downstream activities. Table 2 shows leukemia/lymphoma cell lines, their corresponding diseases and aberrant NKL homeobox gene activities to help identify models to study a particular type of cancer and/or gene of interest.

In summary, NKL homeobox genes are physiologically expressed in hematopoiesis in a unique and specific pattern which we have termed NKL-code. This code serves to identify and evaluate deregulated NKL homeobox genes in leukemia/lymphoma. Aberrant activities of these basic developmental regulators contribute to the pathogenesis of hematopoietic malignancies. The knowledge of their pathophysiological activity may support the design of improved diagnostics and novel therapies in the future. Finally, validated cell lines represent suitable models to study the regulation and function of NKL homeobox genes in normal and malignant hematopoietic cells.

## Figures and Tables

**Table 1:** Aberrantly expressed NKL homeobox genes in leukemia/lymphoma patients. This table lists all 48 NKL homeobox genes which are deregulated in defined lymphoid and myeloid malignancies (totally 35). NKL-code members are indicated by gray background, genes not represented on the analyzed expression arrays are indicated in blue letters. Lymphoid malignancies are diffuse large B-cell lymphoma (DLBCL), follicular lymphoma (FL), hairy cell lymphoma (HCL), Hodgkin lymphoma (HL), mantle cell lymphoma (MCL), and splenic marginal zone lymphoma (SMZL), angioimmunoblastic T-cell lymphoma (AITL), anaplastic large cell lymphoma (ALCL), adult T-cell leukemia/lymphoma (ATLL), hepatosplenic T-cell lymphoma (HSTL), NKT-cell lymphoma (NKTL), peripheral T-cell lymphoma (PTCL), and T-cell acute lymphoid leukemia (T-ALL). B- and T-cell malignancies are indicated in orange. Myeloid malignancies comprise acute myeloid leukemia (AML) and myelodysplastic syndrome (MDS) and are indicated in green.

	DLBCL	FL	HCL	HL	MCL	SMZL	AITL	ALCL	ATLL	HSTL	NKTL	PTCL	T-ALL	AML	MDS
BARHL1															
BARHL2															
BARX1															
BARX2															
BSX															
DBX1															
DBX2															
DLX1															
DLX2															
DLX3															
DLX4															
DLX5															
DLX6															
EMX1															
EMX2															
EN1															
EN2															
HHEX															
HLX															
HMX1															
HMX2															
HMX3															
LBX1															
LBX2															
MSX1															
MSX2															
NANOG															
NKX1-1															
NKX1-2															
NKX2-1															
NKX2-2															
NKX2-3															
NKX2-4															
NKX2-5															
NKX2-6															
NKX2-8															
NKX3-1															
NKX3-2															
NKX6-1															
NKX6-2															
NKX6-3															
NOTO															
TLX1															
TLX2															
TLX3															
VAX1															
VAX2															
VENTX															
48 (35)	3	3	2	6	5	3	7	9	6	6	11	11	24	18	14

**Table 2:** Aberrantly expressed NKL homeobox genes in leukemia/lymphoma cell lines. This table lists hematopoietic cell lines in which particular NKL homeobox genes are overexpressed. Additionally, the corresponding disease and potentially relevant information are given. NKL-code members are indicated by gray background, genes not represented on the analyzed expression array are indicated in blue.

Gene	Cell Line	Disease	Regulated by	Effect, Target Genes	Reference
BARHL1					
BARHL2					
BARX1					
BARX2	RPMI-8226	MM			[113]
BSX					
DBX1					
DBX2					
DLX1	EOL-1	AML			[98]
DLX2	EOL-1	AML	ERK-signaling		[98]
	HPB-ALL	T-ALL			[113]
DLX3					
DLX4					
DLX5	NOMO-1	AML	NANOG		[30]
DLX6	NOMO-1	AML	NANOG		[30]
EMX1					
EMX2					
EN1					
EN2					
HHEX	CCRF-CEM	T-ALL			[113]
	RPMI-8402	T-ALL			[113]
HLX	L-540	HL	aberrant STAT3 activity		[91]
	DOHH-2	DLBCL	EBV-mediated STAT3		[92]
	OCI-LY19	DLBCL			[113]
	NU-DHL-1	DLBCL			[113]
	SEM	BCP-ALL			[113]
	DEL	ALCL	aberrant STAT3 activity		[29]
	KI-JK	ALCL	aberrant STAT3 activity		[29]
	L-82	ALCL	aberrant STAT3 activity		[29]
	SR-786	ALCL	aberrant STAT3 activity		[29]
	SU-DHL-1	ALCL	aberrant STAT3 activity		[29]
	SUP-M2	ALCL	aberrant STAT3 activity		[29]
HMX1					
HMX2	EOL-1	AML		inhibits EPX, activates HRT7 and ERK	[98]
	MOLM-13	AML			[98]
	MV4-11	AML			[98]
	697	BCP-ALL			[98]
	REH	BCP-ALL			[98]
HMX3	EOL-1	AML		inhibits EPX, activates HRT7 and ERK	[98]
	MOLM-13	AML			[98]
	MV4-11	AML			[98]
	SEM	BCP-ALL			[113]
LBX1					
LBX2					
MSX1	L-1236	HL	suppressed BMP-pathway	cofactor H1C, target ZHX2	[114]
	GRANTA-519	MCL		CCND1 in t(11;14)(q13;q32)	[88]
	JEKO-1	MCL		CCND1 in t(11;14)(q13;q32)	[88]
	REC-1	MCL		CCND1 in t(11;14)(q13;q32)	[88]
	LOUCY	ETP-ALL			[89]
MSX2					
NANOG	NOMO-1	AML		NOTCH, CDK6, MIR17HG	[30]
NKX1-1					
NKX1-2					
NKX2-1	SU-DHL-5	DLBCL	KMT2A, H2B		[83]
	RPMI-8402	T-ALL			[113]
NKX2-2	DEV	HL	IL17RB-signaling		[115]
NKX2-3					
NKX2-4					
NKX2-5	CCRF-CEM	T-ALL	t(5;14)(q35;q32)	activates MEF2C	[76,80]
	PEER	T-ALL	t(5;14)(q35;q32)	activates MEF2C	[76,80]
NKX2-6					
NKX2-8					
NKX3-1	JURKAT	T-ALL	TAL1 and GATA3	activates SIX6	[84,86]
	MOLT-4	T-ALL			[86]
	PER-117	T-ALL			[86]
	PF-392	T-ALL			[86]
	RPMI-8402	T-ALL			[86]
	OCI-LY9	DLBCL			[113]
NKX3-2	CCRF-CEM	T-ALL	suppressed BMP-pathway	activates SIX6	[87]
NKX6-1					
NKX6-2					
NKX6-3	DOHH-2	DLBCL	repressed by HLX and HHEX, activated by PAX5 and MYB	inhibits PAX5 and MSX1	[27]
NOTO					
TLX1	ALL-SIL	T-ALL	t(10;14)(q24;q11)	ETS1-interaction	[70,75]
TLX2	SUP-HD1	HL	activated by PBX1	activates TBX15	[45]
TLX3	DND-41	T-ALL	t(5;14)(q35;q32)		[116]
	HPB-ALL	T-ALL	t(5;14)(q35;q32)		[117]
VAX1					
VAX2					
VENTX	SEM	BCP-ALL			[113]
